# A nutrient profiling system for the (re)formulation of a global food and beverage portfolio

**DOI:** 10.1007/s00394-016-1161-9

**Published:** 2016-02-15

**Authors:** Antonis Vlassopoulos, Gabriel Masset, Veronique Rheiner Charles, Cassandra Hoover, Caroline Chesneau-Guillemont, Fabienne Leroy, Undine Lehmann, Jörg Spieldenner, E-Siong Tee, Mike Gibney, Adam Drewnowski

**Affiliations:** 1 Nestlé, Nutrient Profiling, Public Health Nutrition, Nestlé Research Center, Vers-chez-les-Blanc, P.O. Box 44, 1000 Lausanne 26, Switzerland; 2 Nestlé USA, 30003 Bainbridge Road, Solon, OH 44120 USA; 3 Nestlé France, 7 Boulevard Pierre Carle, 77186 Noisiel, France; 4 TES NutriHealth Strategic Consultancy, c/o 46, Jalan SS 22/32, 47400 Petaling Jaya, Selangor Malaysia; 5 Institute of Food and Health, University College Dublin, Belfield, Dublin 4, Ireland; 6 School of Biomedical Sciences, University of Ulster, Coleraine, UK; 7 Center for Public Health Nutrition, University of Washington, Box 353410, Seattle, WA 98195-3410 USA

**Keywords:** Nutrition, Foods, Nutrient profiling, Reformulation

## Abstract

**Purpose:**

To describe the Nestlé Nutritional Profiling System (NNPS) developed to guide the reformulation of Nestlé products, and the results of its application in the USA and France.

**Design:**

The NNPS is a category-specific system that calculates nutrient targets per serving as consumed, based on age-adjusted dietary guidelines. Products are aggregated into 32 food categories. The NNPS ensures that excessive amounts of nutrients to limit cannot be compensated for by adding nutrients to encourage. A study was conducted to measure changes in nutrient profiles of the most widely purchased Nestlé products from eight food categories (*n* = 99) in the USA and France. A comparison was made between the 2009–2010 and 2014–2015 products.

**Results:**

The application of the NNPS between 2009–2010 and 2014–2015 was associated with an overall downwards trend for all nutrients to limit. Sodium and total sugars contents were reduced by up to 22 and 31 %, respectively. Saturated Fatty Acids and total fat reductions were less homogeneous across categories, with children products having larger reductions. Energy per serving was reduced by <10 % in most categories, while serving sizes remained unchanged.

**Conclusions:**

The NNPS sets feasible and yet challenging targets for public health-oriented reformulation of a varied product portfolio; its application was associated with improved nutrient density in eight major food categories in the USA and France. Confirmatory analyses are needed in other countries and food categories; the impact of such a large-scale reformulation on dietary intake and health remains to be investigated.

## Introduction

Improving the nutrient density of food products through reformulation is one approach to improve diet quality and to reduce the prevalence of non-communicable diseases (NCDs) [1, 2]. Food reformulation can be either mandatory or voluntary, and it has been identified as one of the most relevant and cost-effective public health nutrition strategies [3]. The World Health Organization (WHO) and the World Health Assembly have identified the need for the food industry to reduce the amounts of saturated and trans-fat, free sugars and salt in the global food supply [4]. Focusing on the overall nutrient density of foods, the WHO European Action Plan emphasized that new nutrient-rich food products would need to be developed to achieve dietary goals at the population level [5].

Nutrient profiling is described as the science of ranking or classifying foods based on their nutrient composition for the purpose of preventing disease and promoting health [6]. Nutrient profiling models can be used for various applications, including the regulation of nutrition and health claims [7, 8], marketing of food products to children [9, 10], product promotion at point of sale [11], and front-of-package labeling [12]. For the food industry, an important application of nutrient profiling is to assist in developing a more holistic, nutrition-oriented reformulation of their food portfolio [13, 14].

Multiple nutrient profiling models are currently available; while most are in the public domain [7–9, 15], some are proprietary [11]. These models tend to vary widely in their selection of nutrients, the basis used for calculation (per 100 g/kcal or per serving), and the choice of thresholds or scoring algorithm used [15]. In “across-the-board” models, the same algorithm is applied to all foods and beverages, whereas “category-specific” models use different algorithms for different food categories [14]. Regardless of the parameters employed by each model, nutrient profile models need to be specifically designed for the purpose for which they will be used. Front-of-pack labeling systems were shown to have some effect on product reformulation [16], but the literature on evaluating the profiling systems specifically developed for reformulation and their performance is limited [14].

The Nestlé Nutritional Profiling System (NNPS) was designed specifically for the reformulation of a global product portfolio spanning several food and beverage categories, taking into consideration both the consumers’ eating habits and the needs of specific age groups. The objective of this paper is to present the NNPS and changes in nutrient density across eight food categories in the USA and France following the application of the NNPS between 2009–2010 and 2014–2015.

## Materials and methods

### Scope and principles of the Nestlé Nutritional Profiling System (NNPS)

Our goal was to develop a nutrient profiling system to help guide the formulation of new foods and beverages or the reformulation of existing ones. This methodology is being implemented for the majority of the Nestlé food and beverage portfolio. Infant formulas and medical nutrition products are regulated separately and are beyond the scope of the NNPS, as are food products for children <4 years of age. The NNPS was only applied to the brands owned by Nestlé; the products sold from Cereal Partners Worldwide (a joint venture between Nestlé and General Mills) were not profiled with the NNPS but with the specific profiling system used by the joint venture [17].

The outcome of the NNPS is dichotomous, i.e., “YES” or “NO,” and relies on pre-defined target values for a set of nutritional factors. To achieve a positive outcome, all target values must be met, i.e., the algorithm is non-compensatory. Four guiding principles were used to define targets for the nutritional factors: (1) nutritional factors include energy, nutrients to limit and nutrients to encourage; (2) the set of nutritional factors and associated target values is category specific; (3) target values are age specific, depending on the products; and (4) target values are defined per serving of the product as consumed.

Pure roast and ground coffee, soluble coffee, pure tea, plain water and products consisting of >95 % whole milk with no added energy-providing nutrients were identified as not in the scope of reformulation for nutrients to limit and are therefore scored as “YES.”

The system, originally developed in 2004, has a dynamic design that allows for the product categories, nutrient targets and reference values to be modified to incorporate the latest evidence in the area of food technology and public health nutrition. Here, we present the latest version of the system as modified in March 2014.

### Development of product categories

The NNPS food categorization was decided by a cross-functional team that included food technologists and nutritionists and taking into account the renovation history of the food category as well as latest innovations in food science. The food categorization process was structured in two steps: (1) identifying the product role in the diet and (2) grouping products based on similar nutrient composition and/or challenges in the reformulation process.

In the first step, the role of the product in the daily diet was considered. The total daily energy intake was divided into the different eating occasions. Based on the results of a literature review of dietary guidelines and dietary intake surveys [18], the NNPS assumes an energy repartition pattern of three main meal occasions, each accounting for 20–35 % of daily energy intake, and 1–2 snacking occasions, each accounting for 5–10 % of daily energy intake. Three main product roles were identified from these eating occasions: larger meal components, smaller meal components/snacks, and accessories. Larger meal components represent foods that provide the main source of energy during one of the three main meals. Smaller meal components and snacks are products either consumed in-between meals or as part of one of the main meals, but in the latter case they are normally consumed as an appetizer, dessert or individual element of a dish providing less than one-third of the overall energy from the meal. Accessories are products consumed as a minor component of a meal (cold sauces, dressings) or between meals (e.g., hard candies) and share the common characteristic of very small energy contribution to the daily diet.

The second step in defining food categories aimed at pooling together products based on those parameters significantly influencing a product’s nutrient composition. This allowed for the establishment of challenging yet achievable targets. Such grouping was either:

A. Ingredient based, i.e., based on the presence of a dominant raw material or of a specific combination of raw materials (cereal- based, milk- based, cocoa- based -  e.g., chocolate - , vegetable based - e.g., soups-, or a specific combination - e.g., pizzas)
B. Process based, i.e., products sharing the same unique production process impacting the nutritional composition (e.g., ice creams, cheeses). In the case of ice cream and cheese the key ingredient is dairy milk/cream in both, but technological constraints are different due to the role of salt in cheese for ripening and preservation versus the role of sugar in the freezing of ice cream.
C. Combination of the above, i.e., water ice and sorbet share common production considerations with ice cream (freezing), but they differ in the principal ingredient (dairy vs. water/juice based).

Categories relevant to the Nestlé food and beverage portfolio were considered; this included the majority of manufactured foods and beverages available in the food supply. The categories were named in such a way that food developers could easily identify which Nestlé products would fall into a specific category (e.g., dairy-based accessories were named as sweetened condensed milk based on the products available in the Nestlé portfolio). The resulting food categories are listed in Table 1.

**Table 1:** Classification of food items into groups and categories, and the corresponding list of nutrients to limit and nutrients to promote per category (*n* = 32)

Food groups	Food categories	Range of Kcal/serving	Rationale for food category setting	Nutrients to limit	Nutrients to encourage
Larger meal components	Milk-based breakfast beverages	A: ≤300 kcal/serving C: ≤255 kcal/serving	Ingredient based	E, TF,SFA,TFA,AS,Fr,Na	P,Ca
	Cereal-based foods	A: 200–400 kcal/serving C: 170–340 kcal/serving	Ingredient based	E, TF,SFA,TFA,AS,Fr,Na	P,Ca,F
	Complete meals (all dishes eaten as main part of meal including pizzas >185 g)	A: ≤600 kcal/serving C: ≤510 kcal/serving		E, TF,SFA,TFA,AS,Fr,Na	P + 1 from list*
	Side dishes and center of plate food items	A: ≤400 kcal/serving C: ≤340 kcal/serving		E, TF,SFA,TFA,AS,Fr,Na	P + 1 from list*
	Asian noodles as main dish	A: ≤600 kcal/serving C: ≤510 kcal/serving	Processed based	E, TF,SFA,TFA,AS,Fr,Na	–
	Pizza as center of plate (<185 g)	A: ≤400 kcal/serving C: ≤340 kcal/serving	Ingredient based	E, TF,SFA,TFA,AS,Fr,Na	P +1 from list*
Smaller meal components/snacks	Soups	A: ≤200 kcal/serving C: ≤170 kcal/serving	Ingredient based	E, TF,SFA,TFA,AS,Fr,Na	–
	Cold cuts and spreads	A: ≤200 kcal/serving C: ≤170 kcal/serving	Ingredient based	E, TF,SFA,TFA,AS,Fr,Na	–
	Breads and pizza doughs	A: ≤200 kcal/serving C: ≤170 kcal/serving	Ingredient based Process based	E, TF,SFA,TFA,AS,Fr,Na	F
	Savory snacks	A: ≤200 kcal/serving C: ≤170 kcal/serving	Ingredient based	E, TF,SFA,TFA,AS,Fr,Na	–
	Salty and savory biscuits	A: ≤200 kcal/serving C: ≤170 kcal/serving	Ingredient based Process based	E, TF,SFA,TFA,AS,Fr,Na	–
	Cheeses	A: ≤200 kcal/serving C: ≤170 kcal/serving	Ingredient based Process based	E, TF,SFA,TFA,AS,Fr,Na	P
	Yogurts and fresh cheeses	A: ≤200 kcal/serving C: ≤170 kcal/serving	Ingredient based Process based	E, TF,SFA,TFA,AS,Fr,Na	P,Ca
	Dairy desserts	A: ≤200 kcal/serving C: ≤170 kcal/serving	Ingredient based	E, TF,SFA,TFA,AS,Fr,Na	Ca
	Ice creams	A: ≤200 kcal/serving C: ≤170 kcal/serving	Process based	E, TF,SFA,TFA,AS,Fr,Na	–
	Water ices & sorbets	A: ≤100 kcal/serving C: ≤85 kcal/serving	Ingredient based Process based	E, TF,SFA,TFA,AS,Fr,Na	–
	Enriched beverages	A: ≤200 kcal/serving C: ≤170 kcal/serving	Ingredient based	E, TF,SFA,TFA,AS,Fr,Na	P + 1 from list*
	Culinary sauces	A: ≤150 kcal/serving C: ≤127.5 kcal/serving	Ingredient based	E, TF,SFA,TFA,AS,Fr,Na	–
	Milk-based beverages for consumption as small part of a meal or in-between meals	A: ≤200 kcal/serving C: ≤170 kcal/serving	Ingredient based	E, TF,SFA,TFA,AS,Fr,Na	P,Ca
	Cereal-based snacks, cereal-based products for consumption as small part of a meal or in-between meals	A: ≤200 kcal/serving C: ≤170 kcal/serving	Ingredient based	E, TF,SFA,TFA,AS,Fr,Na	F
	Confectionary bars (non-chocolate based)	A: ≤200 kcal/serving C: ≤170 kcal/serving	Ingredient based Process based	E, TF,SFA,TFA,AS,Fr,Na	–
	Chocolate	A: ≤200 kcal/serving C: ≤170 kcal/serving	Ingredient based	E, TF,SFA,TFA,AS,Fr,Na	–
	Juice-based beverages	A: ≤200 kcal/serving C: ≤170 kcal/serving	Ingredient based	E, TF,SFA,TFA,AS,Fr,Na	–
	Cakes, cookies and desserts	A: ≤200 kcal/serving C: ≤170 kcal/serving	Ingredient based Process based	E, TF,SFA,TFA,AS,Fr,Na	–
Accessories	Beverages (all beverages with <2 % milk protein or <50 % juice or <25 % cereal on dry basis)	A: ≤100 kcal/serving C: ≤85 kcal/serving	Ingredient based	E, TF,SFA,TFA,AS,Fr,Na	–
	Sugar confectionary	A: ≤100 kcal/serving C: ≤85 kcal/serving	Ingredient based	E, TF,SFA,TFA,AS,Fr,Na	–
	Sweetened condensed milk	A: ≤100 kcal/serving C: ≤85 kcal/serving	Ingredient based	E, TF,SFA,TFA,AS,Fr,Na	–
	Dressings	A: ≤100 kcal/serving C: ≤85 kcal/serving	Ingredient based	E, TF,SFA,TFA,AS,Fr,Na	–
	Mayonnaise	A: ≤100 kcal/serving C: ≤85 kcal/serving	Ingredient based	E, TF,SFA,TFA,AS,Fr,Na	–
	Cold sauces	A: ≤100 kcal/serving C: ≤85 kcal/serving	Ingredient based	E, TF,SFA,TFA,AS,Fr,Na	–
	Bouillons	A: ≤100 kcal/serving C: ≤85 kcal/serving	Process based	E, TF,SFA,TFA,AS,Fr,Na	–
	Culinary sauces as accessory (<100 mL)	A: ≤100 kcal/serving C: ≤85 kcal/serving	Ingredient based	E, TF,SFA,TFA,AS,Fr,Na	–

*A* adult >11 years old; *C* children 4–11 years old; *E* energy; *TF* total fat; *SFA* saturated fatty acids; *TFA* trans-fatty acids; *AS* added sugars; *Fr* fructose; *Na* sodium; *P* protein; *Ca* calcium; *F* fiber

* +1 from list: requirement achieved when the adequate level of one nutrient/ingredient in the following list is meat:vegetables/fruits (≥80 g/serving), whole grains (≥16 g/serving), vitamins/minerals (≥10 %DV/serving or 5 %DV/100 kcal), essential fatty acids ALA (n3): >0.6 % of energy, LA (n6): 5–10 % of energy

### Selection of nutritional factors

Based on the World Health Assembly Global Strategy on Diet, Physical Activity and Health [4], saturated fatty acids (SFA), trans-fatty acids (TFA), added sugars (AS) and sodium (Na) were designated as compulsory nutrients to limit across all categories. The NNPS is a system designed to be equally applicable for use by food developers and nutritionists; to facilitate optimal guidance for the end user, additional factors were incorporated into the system. Under this scope, three additional factors were added for these respective reasons: total fat (TF) to ensure the overall reduction in fat content alongside the removal of SFA and TFA, energy (E) to guide portion size optimization and fructose (Fr) to ensure that fructose or high-fructose corn syrup (HFCS) (ingredients with higher sweetening power) were not used excessively in light of any possible health effects of high-fructose consumption [19] (Table 1). Only TFA originating from partially hydrogenated oils were considered in the calculation of nutrient profiles, in line with the company’s policy on trans-fat [20]. Added sugars were defined as all mono- and disaccharides added during the manufacturing or preparation of a product. Naturally occurring sugars (lactose from milk/dairy fractions, mono- and disaccharides from unsweetened fruit ingredients) were excluded provided that the unsweetened fruit ingredient was not added for sweetening purposes. Based on the molecular structure, any added sucrose was counted as 50 % fructose and 50 % glucose. For the calculation of sodium, both added sodium and sodium from natural sources were considered.

Alongside the nutrients to limit, a list of nutrients to encourage was identified (e.g., protein, calcium, fiber). Targets for nutrients to encourage were included in product categories where this was considered sensible and where the nutrients supported the role of the product in the diet (Table 1). All products falling under the larger meal components group were required to include at least one nutrient to encourage, as they are the main energy sources of the diet. Due to their importance in the diet, the categories complete meals, and side dishes & center of plate food items (i.e., food items acting as the principal protein source in a main meal) needed to be profiled against a minimum of two nutrients to encourage, including protein. In such cases, the second compulsory nutrient to encourage had to be selected from a list of nutrients/ingredients. For milk and dairy products, protein and calcium were considered nutrients to encourage. Fiber was considered a nutrient to encourage for cereal-based foods. The list of other nutrients/ingredients to encourage included fruits & vegetables, whole grains, vitamins, minerals and essential fatty acids all selected according to the relevance for the specific food product (Table 1). No nutrients to encourage were applied to products classified as accessories, because their contribution to the diet was considered too small.

### Selection of age-appropriate daily reference values

Daily reference values (DVs) of various nutrients to encourage and various nutrients to limit are presented in Table 2. Consumers were split into three age groups: adults, children 4–8 years old and children 9–11 years old (children >11 years old were considered as “adults”). Public health nutritional recommendations for each age group were applied to define what constitutes a nutritionally adequate diet.

**Table 2:** Daily reference values (DVs) of various nutrients to encourage and nutrients to limit, for adults and children [14, 21–30]

Nutrient	DVs for children aged 4–8 years	DVs for children aged 9–11 years	DVs for adults
Energy	1700 kcal	2000 kcal	2000 kcal
*Nutrients to limit*			
Total fat	60 g	70 g	70 g
Saturated fat	19 g	20 g	20 g
Trans-fat	<1 % total energy	<1 % total energy	<1 % total energy
Added sugar^a^	42.5 g	50 g	50 g
Sodium	1400 mg	2000 mg	2400 mg
*Nutrients to encourage*			
Protein	24 g	50 g	50 g
Dietary fiber	15 g	17 g	25 g
Calcium	700 mg	1000 mg	1000 mg
Iron	14 mg	14 mg	9 mg
Iodine	150 μg	150 μg	100 μg
Magnesium	300 mg	300 mg	100 mg
Zinc	15 mg	15 mg	11 mg
Vitamin A	800 μg	800 μg	500 μg
Thiamin	1.2 mg	1.2 mg	0.9 mg
Riboflavin	1.2 mg	1.2 mg	0.9 mg
Niacin	15 mg	15 mg	12 mg
Vitamin B6	1.3 mg	1.3 mg	1 mg
Folic acid	240 μg	240 μg	300 μg
Vitamin B12	2.4 μg	2.4 μg	1.8 μg
Vitamin C	60 mg	60 mg	35 mg
Vitamin D	5 μg	5 μg	5 μg

^a^Includes mono- and disaccharides present in the raw materials such as sugar, corn syrup, corn syrup solids, fructose sweetener, honey, molasses and all powdered form of any syrup. Naturally occurring sugars (lactose from milk/dairy fractions, mono- and disaccharides from unsweetened fruit ingredients) are excluded provided that the unsweetened fruit ingredient is not added for sweetening purposes

The United States Food and Drug Administration (USFDA) DVs [21] in combination with the Institute of Medicine (IOM) recommendations for total fat intake [22] were used as the initial basis to set the NNPS global DVs for a 2000 kcal daily diet for adults. In the absence of specific US FDA values for trans-fat and added sugars, the WHO recommendations were followed [23, 24]. In each NNPS update the latest recommendations of the WHO and other bodies were considered in order to adjust the DVs. DVs for children were adopted accordingly for a 1700 kcal daily diet for children 4–8 years old based on the IOM recommendation [22]. For children, dietary fiber intake followed the European Food Safety Authority (EFSA) guidance [25], while for all micronutrients, except sodium, adult DVs followed CODEX recommendations [26] and children DVs followed recommendations of the Joint FAO/WHO Expert Consultation [27]. NNPS is applied globally through a dedicated proprietary software which allows local DVs to be applied as the basis of the profiling system instead of using international DVs, when the former are available. This decision was made to allow the system to address local dietary needs.

### Selection of the base for calculation

Nutritional factor targets were set based on the assumption that food products are consumed as part of a nutritionally adequate diet [28]. As a result, the base for calculation was decided to be one serving of the final product as consumed after reconstitution and/or cooking. Consumer behavior data from local market research were used to define the eating habits and estimate amounts customarily consumed as portion sizes in each geographical region separately. To help guide the reformulation of serving, maximum energy targets per serving were developed for each food category.

### Development of nutrient and energy targets per category

Following the principles described below, target values were set for energy, nutrients to limit and nutrients to encourage (Tables 3, 4).

**Table 3:** Nutrient targets for nutrients to limit per product category

	Energy	Total fat	Saturated fatty acids	Trans-fatty acids	Added sugars	Fructose	Sodium
	%DV/serving	As indicated, per serving	As indicated, per serving	% tot fat	As indicated, per serving	% added sugars	%DV/serving
*Larger meal components*							
Milk-based breakfast beverages	≤15	≤10 %DV or ≤30 %en ≤50 %en in children^a^	≤20 %DV or ≤15 %en ≤65 %TF in children^a^	≤2	≤25 %DV or ≤25 %en	≤50	≤10 or ≤5/100 kcal
Cereal-based foods	10–20	≤30 %en ≤50 %en in children^a^	≤15 %en ≤65 %TF in children^a^	≤2	≤25 %en	≤50	≤5/100 kcal
Complete meals (all dishes eaten as main part of meal including pizzas >185 g)	≤30	≤35 %en	≤15 %DV	≤2	≤25 %en	≤50	≤40
Side dishes and center of plate foods*	≤20	≤15 %DV or ≤40 %en	≤20 %DV or ≤20 %en	≤2	≤15 %DV or ≤15 %en	≤50	≤25
Asian noodles as main dish	≤30	≤35 %en	≤15 %en	≤2	≤25 %en	≤50	≤40
Pizza as center of plate (<185 g)	≤20	≤15 %DV or ≤40 %en	≤17.5 %DV or ≤17.5 %en	≤2	≤10 %DV or ≤10 %en	≤50	≤33
*Smaller meal components/snacks*							
Soups	≤10	≤7.5 %DV	≤7.5 %DV	≤2	≤5 %DV	≤50	≤33
Cold cuts and spreads	≤10	≤10 %DV	≤10 %DV	≤2	≤5 %DV	≤50	≤10
Breads and pizza doughs	≤10	≤10 %DV	≤10 %DV	≤2	≤5 %DV	≤50	≤10
Savory snacks	≤10	≤10 %DV	≤10 %DV	≤2	≤5 %DV	≤50	≤12.5
Salty and savory biscuits	≤10	≤15 %DV	≤15 %DV	≤2	≤5 %DV	≤50	≤12.5
Cheeses	≤10	≤10 %DV ≤50 %en in children^a^	≤20 %DV ≤65 %TF in children^a^	≤2	≤5 %DV	≤50	≤15
Yogurts and fresh cheeses	≤10	≤7.5 %DV ≤50 %en in children^a^	≤15 %DV ≤65 %TF in children^a^	≤2	≤25 %DV	≤50	≤10
Dairy desserts	≤10	≤10 %DV ≤50 %en in children^a^	≤20 %DV ≤65 %TF in children^a^	≤2	≤25 %DV	≤50	≤10
Ice creams	≤10	≤15 %DV	≤20 %DV	≤2	≤25 %DV	≤50	≤5
Water ices and sorbets	≤5	≤5 %DV	≤5 %DV	≤0.5^b^	≤25 %DV	≤50	≤5
Enriched beverages	≤10	≤10 %DV	≤10 %DV	≤2	≤25 %DV	≤50	≤10
Culinary sauces	≤7.5	≤7.5 %DV	≤10 %DV	≤2	≤5 %DV	≤50	≤17.5
Milk-based beverages for consumption as small part of a meal or in-between meals	≤10	≤10 %DV ≤50 %en in children^a^	≤20 %DV ≤65 %TF in children^a^	≤2	≤25 %DV	≤50	≤10
Cereal-based snacks/products for consumption as small part of a meal or in-between meals	≤10	≤7.5 %DV	≤15 %DV	≤2	≤25 %DV	≤50	≤10
Confectionary bars (non-chocolate based)	≤10	≤10 %DV	≤10 %DV	≤2	≤25 %DV	≤50	≤5
Chocolate	≤10	≤15 %DV	≤20 %DV or ≤65 %TF	≤2	≤25 %DV	≤50	≤5
Juice-based beverages	≤10	≤5 %DV	≤5 %DV	≤2	≤1 %DV	≤50	≤5
Cakes, cookies and desserts	≤10	≤15 %DV	≤15 %DV	≤2	≤25 %DV	≤50	≤7.5
*Accessories*							
Beverages (all beverages with <2 % milk protein or <50 % juice or <25 % cereal on dry basis)	≤5	≤7.5 %DV	≤10 %DV	≤2	≤25 %DV	≤50	≤5
Sugar confectionary	≤5	≤5 %DV	≤5 %DV	≤2	≤25 %DV	≤50	≤5
Sweetened condensed milk	≤5	≤5 %DV ≤50 %en in children^a^	≤10 %DV ≤65 %TF in children^a^	≤2	≤25 %DV	≤50	≤5
Dressings	≤5	≤10 %DV	≤5 %DV	≤2	≤5 %DV	≤50	≤10
Mayonnaise	≤5	≤17.5 %DV	≤5 %DV	≤2	≤5 %DV	≤50	≤5
Cold sauces	≤5	≤10 %DV	≤5 %DV	≤2	≤5 %DV	≤50	≤5
Bouillons	≤5	≤5 %DV	≤5 %DV	≤2	≤5 %DV	≤50	≤33
Culinary sauces as accessory (<100 mL)	≤5	≤5 %DV	≤7.5 %DV	≤2	≤5 %DV	≤50	≤12.5

*DV* daily value, based on international or local recommendations; *%en* % energy; *%TF* % of total fat

* Center of plate foods describes all the food items that are the main protein carrier of the meal, including fish and meat products as well as plant-based protein sources (like pulses) in the case of vegetarian meals

^a^Children aged 4–11 years

^b^Water ice and sorbet are considered TFA free; hence, the nutrient target is adapted accordingly

**Table 4:** Nutrient targets for compulsory nutrients to promote per product category

Item	%DV/serving		
	Protein	Calcium	Fiber
*Larger meal components*			
Milk-based breakfast beverages	≥10 % and ≥12 %en	≥20 % and ≥111 mg/100 kcal^a^	–
Cereal-based foods	≥10 % and ≥12 %en	≥20 % and ≥111 mg/100 kcal^a^	≥10 %
Complete meals (all dishes eaten as main part of meal including pizzas >185 g)	≥12 %en	–	–
Side dishes and center of plate foods	≥15 % and ≥20 %en	–	–
Asian noodles as main dish	–	–	–
Pizza as center of plate (<185 g)	≥10 % and ≥12 %en	–	–
*Smaller meal components/snacks*			
Breads and pizza doughs	–	–	≥10 %
Cheeses	≥12 %en	–	–
Yogurts and fresh cheeses	≥12 %en	≥111 mg/100 kcal^a^	–
Dairy desserts	–	≥5 %DV/100 kcal^b^	–
Enriched beverages	≥5 % DV/100 kcal		
Milk-based beverages for consumption as small part of a meal or in-between meals	≥12 %en	≥111 mg/100 kcal^a^	–
Cereal-based snacks, cereal-based products for consumption as small part of a meal or in-between meals	–	–	≥5 % or 5 %DV/100 kcal^b,c^

*%en* % energy; *DV* daily value, based on international or local recommendations

^a^≥16 %DV/100 kcal for children 4–8 years old

^b^≥6 %DV/100 kcal for children 4–8 years old

^c^Cereal-based products consumed as a snack are not considered a high source of fiber, but still a potential carrier product so the compulsory fiber target is set to 5 %DV/serving

Energy targets were developed for each product category based on the energy repartition pattern of three main meals (20–35 % DV for energy each) and two snacks (5–10 % DV for energy each) (Table 3): for larger meal components, the maximum energy target was set between 15 and 30 % of total energy depending on whether the components were the sole source of energy in the meal or not; for smaller meal components and snacks, the energy target was set to ≤10 %; for accessories, the target was set to ≤5 %.

The first approach toward setting targets for nutrients to limit was to set up baseline targets aligned with the energy targets for the product category. For example, product categories delivering ≤10 % of daily energy per serving should not deliver more than 10 % of the DV of a given nutrient to limit per serving. Alternatively, for larger meal components delivering ≥10 % of energy per serving, nutrient targets were aligned as closely as possible with inter- or- national dietary recommendations. For example, the WHO recommends that total fat intake should not exceed 30 % of daily energy, and therefore, the target for total fat in cereal-based foods (as a main meal) was set accordingly. As a result, targets for nutrients to limit were expressed in two ways, either as  % of energy (e.g., total fat ≤30 % energy) or as % DV (e.g., sodium ≤10 % DV/serving). The reference unit as % of energy was used for product categories with a higher contribution to the daily energy intake (i.e., larger meal components) for nutrients providing energy; the reference unit as a % DV was used in all product categories delivering ≤10 % of recommended daily energy per serving, for all nutrients.

Category-specific targets were then set up starting from the above-defined “baseline” targets. The setting-up of category-specific targets considered technological and organoleptic aspects by a cross-functional team including nutritionists and food technologists. The following elements were considered: nutrient content of similar foods in the food supply and existing reformulation targets from other profiling systems (Table 2). As an example, although ice creams are not expected to contribute to more than 10 % of the daily energy, the saturated fat and sodium targets were set to ≤20 % DV/serving (allowing for dairy ingredients, including milk fat) and ≤5 % DV/serving (due to a lower intrinsic concentration of sodium in the raw ingredients), respectively, instead of ≤10 % DV/serving. Other examples of category-specific targets include products with a preferred savory or sweet taste, such as salty and savory biscuits (in the case of sodium), or ice cream, water ice and dairy desserts (products where sugar has technical and taste properties) in the case of added sugars. In the case of sodium targets, they were aligned with technical considerations and local profiling systems (e.g., sodium target for soups in the Heart Foundation Tick Australia) or as described in other nutrient profiling systems [14].

In the case of added sugars, a maximum value applying to all category-specific added sugars target was set at ≤25 % DV per serving for the smaller meal components/snacks and accessories product roles and ≤25 % of total energy for larger meal components. This decision was made based on the WHO recommendation of limiting free sugar intake over a maximum of four “sugary” eating occasions [23] and the US (IOM) recommendation that ≤25 % of energy from added sugars should be consumed to ensure adequate micronutrient intake [22]. Unlike other nutrients, TFA were profiled against one common threshold across all categories (Table 3).

Targets were set in a similar way for nutrients to encourage. For protein, the WHO guidance of 10–15 % of total energy [23] was translated into a target of ≥12 % of energy per serving (Table 3). The nutrient target for calcium was set to ≥111 mg/100 kcal in alignment with the protein target, based on an average calcium-to-protein ratio of 37 mg/g found in cow’s milk. For dairy desserts, the calcium target was set to ≥5 % DV/100 kcal based on the CODEX minimum level to claim “source of calcium” for adult products. For children products, the calcium target was set to ≥6 % DV/100 kcal adapted from the target for adults, taking into consideration the lower total energy intake of children (1700 kcal vs. 2000 kcal). The nutrient target for fiber was aligned with the USFDA criterion for “source of fiber” and set to ≥10 % DV per serving (Table 4) [29]. The micronutrient targets were set in alignment with the local requirements for a product to be classified as “source of” (Table 2).

In relevant product categories, targets for fruit and vegetables and whole grain were set at ≥1/2 to ≥1 portion per serving of food or beverage product depending on the product role. A portion of fruit and vegetables was defined as 80 g of fruit and vegetables. The WHO population nutrient intake goals recommend the consumption of at least 400 g of fruit and vegetables per day [23]. This recommendation, combined with local campaigns for the promotion of consumption of fruit and vegetables (“5-a-day” campaigns), allows defining one portion of fruit or vegetable at 80 g. For whole grain, the US dietary guidelines [30] recommend consuming at least 3 oz equivalent of whole grain per day. One ounce corresponds to 28 g of whole grain product, corresponding to 16 g of pure whole grain which was selected as a portion of whole grain.

For the specific case of milk and dairy products for children as well as cereal consumed with milk, the decision was made for the system to allow for whole milk to be used; as a result, the nutrient targets for total fat and saturated fat were adjusted accordingly.

### Validation and testing

A scientific advisory board was put in place to provide guidance and to ensure the validity of the system. In the interest of testing the consistency and magnitude of nutritional changes associated with the application of the NNPS, a case study was conducted on eight product categories in the USA and France: pizza, milk-based beverages (as a snack), water ice and sorbet, and complete meals for the USA; and children’s ice cream, center of plate food items (main protein carriers), soups and cold sauces for France.

The USA and France were selected, being two major countries for Nestlé in terms of sales. Product categories were selected based on the respective volume of sales (covering 40–50 % of total sales) and data completeness of the nutritional information at the time of analysis (nutritional information for 2014–2015). Categories selected were also representative of the three main product roles as defined by the NNPS (i.e., larger meal components, smaller meal components/snacks, and accessories). For each product category, the 15 most widely purchased Nestlé products were identified, unless a product category included less than 15 products in which case all products available were analyzed. Nutrient profiles were retrieved for the years 2009–2010 (as reference year) and 2014–2015 in a paired manner, based on the availability of a complete dataset, so that longitudinal changes after the NNPS application could be analyzed. For the purpose of the manuscript, analyses were focused on total sugars rather than added sugars. Total sugars content is a regulatory requirement for on-pack labeling, and hence, the accuracy of retrospective data is higher than for added sugars. That in turn ensures higher accuracy when studying longitudinal changes. Added sugars are the only fraction of total sugars that is amenable to reformulation; therefore, the reported changes in added sugars reflect directly changes in total sugars. There was no attempt to reduce e.g., naturally occurring sugars in milk. Portion sizes as indicated on the labels were used.

Given that the sample selected for the analysis is a fixed sample of products and not meant to represent all products under the same category, only descriptive statistics were used to identify changes in key nutrients per product category. All reductions were compared against an arbitrary 10 % cutoff point, with this cutoff being used as a measure of how extensive the reformulation has been. As the same parent recipe may be used to produce more than one product, the decision was made to analyze changes in the composition of the most widely purchased products and not the composition of the parent recipes (i.e., not to merge products based on the parent recipe). This strategy focuses on the impact at the consumer level and not on the number of recipes reformulated, plus it accounts for differences in packaging and usage, which can have an impact on the final amount of product (serving) consumed.

## Results

Overall 99 Nestlé food and beverage products were identified through the screening process. Examples of products identified in each category included chocolate milk powder and ready-to-drink flavored milk in milk-based beverages, ham and sausages in center of plate foods, lasagna and macaroni and cheese in complete meals. In the majority of cases, the 15 most widely purchased Nestlé products were different versions of similar recipes, whereas for pizzas, soups and cold sauces each product was linked to a unique recipe. The unique recipes per product category were: 11 for complete meals, 10 for milk-based beverages, 7 for water ice and sorbets, 3 for children’s ice creams and 6 for center of plate foods.

**Fig. 1:**
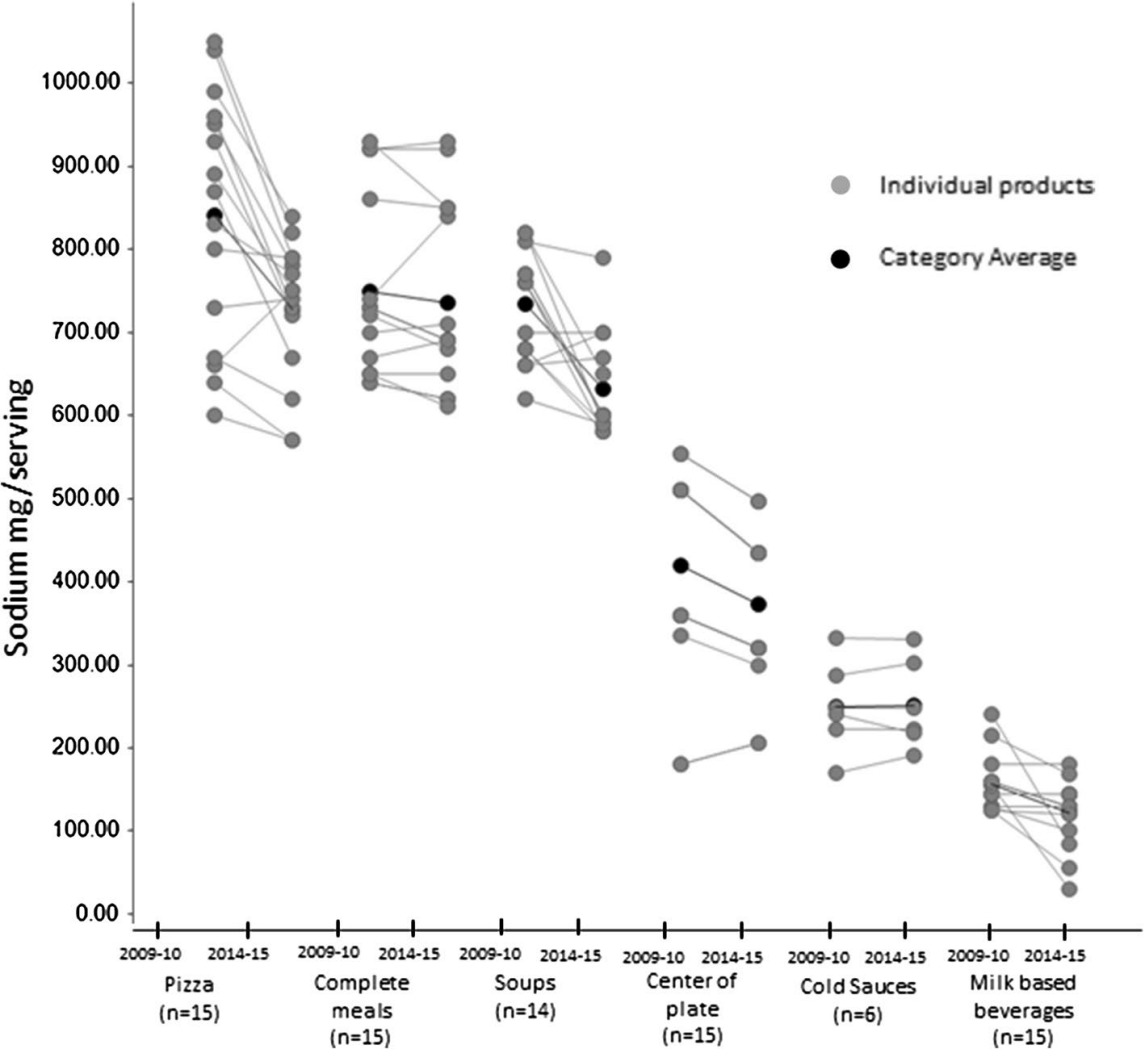
Changes in content per serving for sodium shown for each individual product (*grey*) and the product category average (*black*). *N* represents the number of unique products and not the number of unique recipes. In the figure, points for unique products with similar nutrient profiles are superposed. Also products might have similar content for a given nutrient despite being derived by a different recipe and those are again superposed. Center of plate foods describes all the food items that are the main protein carrier of the meal, including fish and meat products as well as plant-based protein sources (like pulses) in the case of vegetarian meals

**Fig. 2:**
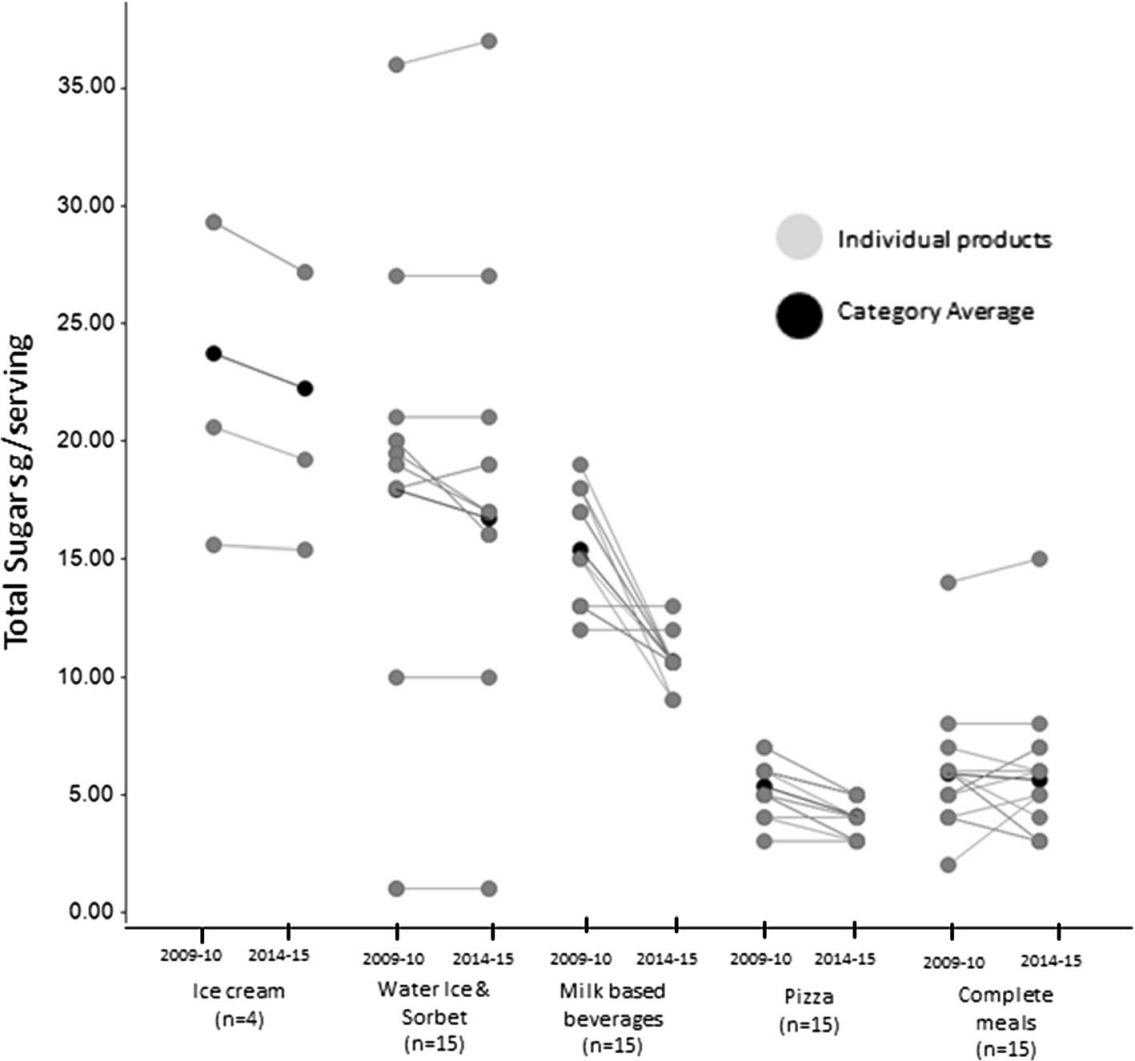
Changes in content per serving for total sugars shown for each individual product (*grey*) and the product category average (*black*). *N* represents the number of unique products and not the number of unique recipes.   In the figure, points for unique products with similar nutrient profiles are superposed. Also products might have similar content for a given nutrient despite being derived by a different recipe and those are again superposed. Center of plate foods describes all the food items that are the main protein carrier of the meal, including fish and meat products as well as plant-based protein sources (like pulses) in the case of vegetarian meals

In all categories combined, the percentage of products meeting all the nutrient targets for the category (classification YES) increased from 36 % in 2009–2010 to 61 % of products in 2014–2015. Among the products classified as NO, 33.3, 46.1, 25.6 and 17.9 % required a further reduction in total sugars, SFA, total fat, and sodium, respectively. Between 2009–2010 and 2014–2015, there was a downward trend in the amounts of all nutrients to limit. Sodium reformulation was homogeneous across almost all categories showing a reduction in sodium content, with the exception of cold sauces which remained stable (increase of less than 2 mg/serving). Sodium reduction was more prominent in products with high sodium content like pizzas, soups and center of plate foods (11–14 % reductions; Fig. 1) as well as children’s products, in this case milk-based beverages (22 % reduction; Table 5). Total sugars were the nutrient with the most extensive reduction of 0.3–4.8 g/serving (Table 6). Milk-based beverages and pizzas had the largest reductions of 31 and 24 %, respectively, with 87 % of all products analyzed having more than 10 % reduction in total sugars content (Table 5). Smaller reductions were seen in complete meals and water ices. The reduction in total sugars in children’s ice creams was the most homogeneous with all products’ contents being reduced by approximately 6 % (Fig. 2).

**Table 5:** Percentage change in key nutrients between 2009–2010 and 2014–2015 in the eight food categories and number of products with reductions ≥10 %

	Serving size		Energy		Total Fat		SFA		Sodium		Total Sugars	
	# Products with ≥10 % reduction *n* (%)	Average change (%)	# Products with ≥10 % reduction *n* (%)	Average change (%)	# Products with ≥10 % reduction *n* (%)	Average change (%)	# Products with ≥10 % reduction *n* (%)	Average change (%)	# Products with ≥10 % reduction *n* (%)	Average change (%)	# Products with≥ 10 % reduction *n* (%)	Average change (%)
Complete meals (*n* = 15)	0 (0)	1	3 (20)	−7	6 (40)	−9	6 (40)	−15	0 (0)	−2	7 (47)	−5
Milk-based beverages (*n* = 15)	0 (0)	0	7 (47)	−16	8 (53)	−36	13 (87)	−66	8 (53)	−22	13 (87)	−31
Water ice and sorbet (*n* = 15)	0 (0)	−2	7 (47)	−8	n/a	n/a	n/a	n/a	n/a	n/a	7 (47)	−7
Pizza (*n* = 15)	0 (0)	−1	1 (7)	−4	4 (27)	−4	8 (53)	−4	9 (60)	−13	13 (87)	−24
Children’s ice cream (*n* = 4)	0 (0)	−2	2 (50)	−9	3 (75)	−25	3 (75)	−20	n/a	n/a	0 (0)	−6
Cold Sauces (*n* = 6)	0 (0)	0	0 (0)	1	2 (33)	−3	2 (33)	−5	0 (0)	1	n/a	n/a
Soups (*n* = 14)	0 (0)	0	0 (0)	6	4 (29)	−15	2 (14)	−13	9 (64)	−14	n/a	n/a
Center of plate (*n* = 15)	0 (0)	1	0 (0)	−1	0 (0)	−2	0 (0)	−2	12 (80)	−11	n/a	n/a

**Table 6:** Average nutrient profiles in 2009–2010 and 2014–2015 for the eight food categories Mean (SD)

	Serving (g)		Energy (kcal)		Total Fat (g)		SFA (g)		Sodium (mg)		Total Sugars (g)	
	2009–10	2014–15	2009–10	2014–15	2009–10	2014–15	2009–10	2014–15	2009–10	2014–15	2009–10	2014–15
Complete meals (*n* = 15)	193.9 (54.3)	195.0 (54.6)	312.7 (30.6)	292.0 (41.4)	12.8 (3.3)	11.6 (3.3)	6.0 (1.8)	5.1 (1.5)	748.7 (106.1)	736.0 (110.4)	5.9 (2.7)	5.6 (3.1)
Milk-based beverages (*n* = 15)	240 (0.0)	240.0 (0.0)	173.3 (16.3)	145.3 (10.6)	2.9 (2.0)	1.9 (1.2)	2.1 (1.2)	0.7 (0.7)	157.0 (32.8)	122.3 (40.0)	15.4 (2.4)	10.6 (1.0)
Water ice (*n* = 15)	80.5 (20.7)	78.9 (20.4)	78.0 (31.8)	71.7 (33.6)	–	–	–	–	–	–	17.9 (8.8)	16.7 (8.8)
Pizza (*n* = 15)	139.9 (10.9)	139.0 (9.5)	341.3 (30.9)	328.7 (33.3)	15.1 (2.4)	14.6 (2.5)	6.5 (1.0)	6.3 (1.3)	840.7 (150.6)	727.3 (84.2)	5.3 (1.1)	4.1 (0.9)
Children's Ice cream (*n* = 4)	77.5 (8.7)	76.3 (6.3)	172.3 (52.7)	157.5 (39.4)	8.7 (2.6)	6.5 (1.7)	5.3 (1.9)	4.2 (1.2)	–	–	23.7 (6.8)	22.3 (5.9)
Cold Sauces (*n* = 6)	8.1 (2)	8.0 (2.2)	37.1 (14.0)	37.4 (14.1)	2.0 (1.3)	2.0 (1.4)	1.3 (0.9)	1.2 (0.9)	250.1 (55.6)	251.8 (54.0)	–	–
Soups (*n* = 14)	242.9 (18.2)	242.8 (18.2)	59.0 (18.5)	62.3 (20.1)	0.8 (0.5)	0.7 (0.5)	0.2 (0.2)	0.1 (0.1)	733.6 (65.7)	631.4 (62.5)	–	–
Center of plate (*n* = 15)	48.0 (11.1)	48.7 (10.1)	96.5 (56.7)	95.4 (54.0)	6.8 (6.1)	6.6 (5.8)	2.7 (2.4)	2.6 (2.2)	420.1 (125.5)	373.1 (94.8)	–	–

**Fig. 3:**
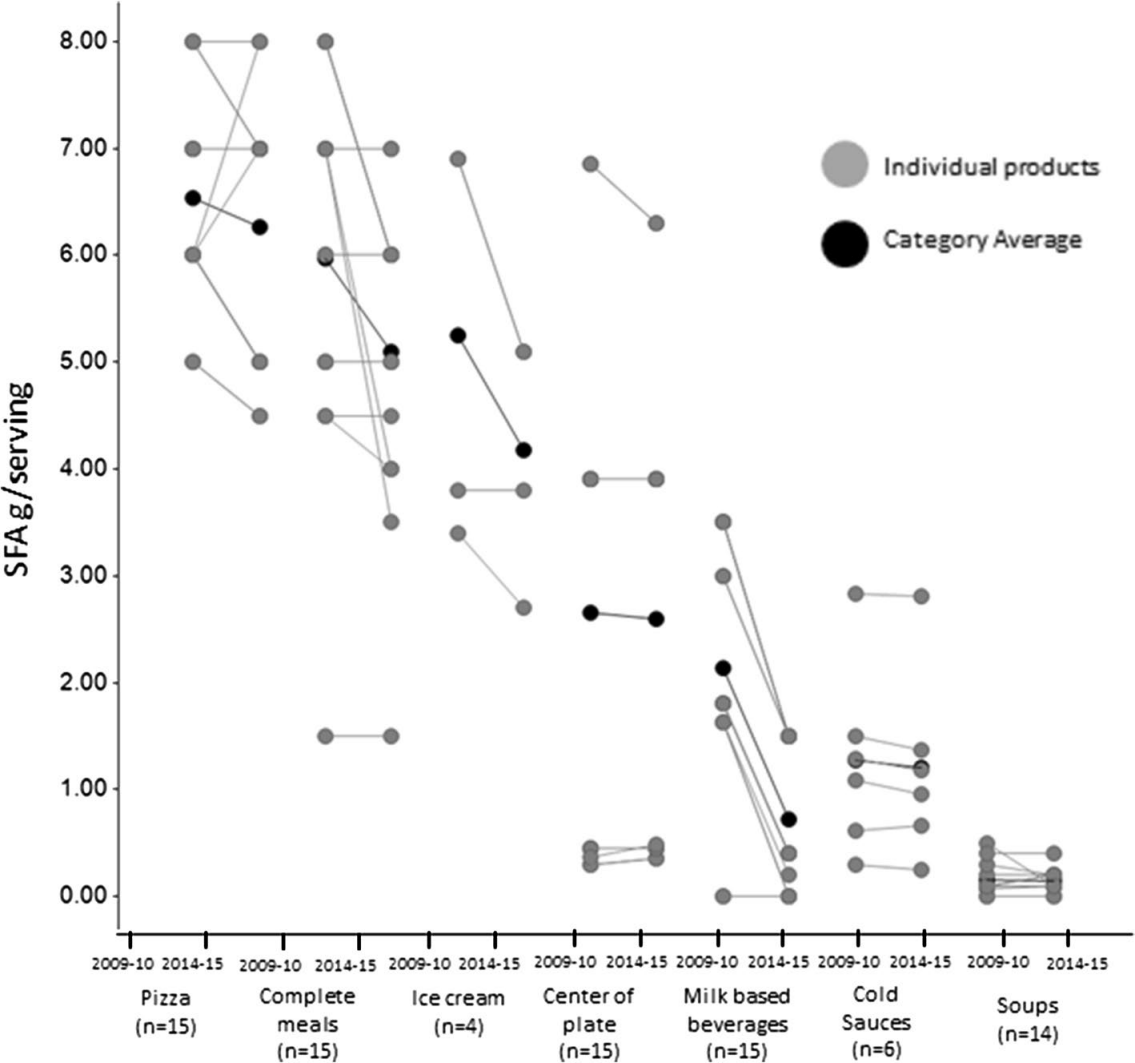
Changes in content per serving for saturated fatty acids shown for each individual product (*grey*) and the product category average (*black*). *N* represents the number of unique products and not the number of unique recipes.   In the figure, points for unique products with similar nutrient profiles are superposed. Also products might have similar content for a given nutrient despite being derived by a different recipe and those are again superposed. Center of plate foods describes all the food items that are the main protein carrier of the meal, including fish and meat products as well as plant-based protein sources (like pulses) in the case of vegetarian meals

Serving sizes were largely unchanged, while reductions were observed in water ices and children’s ice creams in the range of 1.2–1.6 g per serving equivalent to a 2 % reduction (Table 5 and Table 6). Energy per serving was reduced by 1–16 % in 6 out of 8 categories, while small increases in 0.3 and 3.3 kcal/serving were documented in cold sauces and soups, respectively (Table 6). Nearly half of the products in the milk-based beverages, water ices, and children’s ice cream categories had a minimum of 10 % reduction in energy per serving (Table 5). In all categories included there was a reduction in total fat and SFA content. Milk-based beverages had the largest reductions in total fat and SFA (36 and 66 % reductions). Products targeted to children had the largest reductions in total fat (1 g/serving or 36 % in milk-based beverages and 1.2 g/serving or 25 % in children’s ice cream; Tables 5, 6). Reformulation with respect to SFA content had a high variability (Fig. 3), with a main focus on children products followed by soups and complete meals. Pizzas was one product category where some rare cases (*n* = 2) of SFA content increasing were documented (Fig. 3), but at the same time 53 % of all pizza products had SFA reductions larger than 10 % of the original content. Other categories included soups and center of plate foods, but overall only 9 % of the products analyzed showed an increase in SFA, and in the majority of cases, this was linked with an increase in serving size.

## Discussion

The World Health Assembly 2004 specifically called the food industry to set about the reformulation of food products in order to provide affordable, healthy and nutritious choices to consumers. Specifically, the report stated the need for “Initiatives by the food industry to reduce the fat, sugar and salt content of processed foods and portion sizes, to increase introduction of innovative, healthy, and nutritious choices” [4]. This call was renewed by the WHO Plan of action for Europe for the years 2007–2012 [5], and it was also taken up by the EU Platform on Diet, Physical Activity and Health, which put reformulation in its core activities [31]. It is against this background that the NNPS has been established. The present paper sets out to illustrate the challenges that lie behind product reformulation based on a transparent nutrition profiling system. This has been done with 99 food products in eight food categories. This process of reformulation will be both ongoing and global in its reach.

In the USA and France, the majority of products in the eight food categories analyzed showed an improved nutritional content during the 4- to 5-year application of the NNPS. This reformulation effort was associated with lower levels of nutrients to limit, especially sodium and total sugars. Results for SFA and total fat were more category specific but still indicated an overall reduction trend. It is important to highlight that the majority of these changes have been achieved without substantial reductions in serving sizes and with priority given to children’s products.

The NNPS is a compulsory step in product reformulation within the company, and it is the main tool for product developers to improve the product portfolio. The system allows for comparison with other profiling systems or with competitor products against NNPS criteria. In that context, the systematic application of NNPS as a global tool is strongly related to the majority of the changes in the product portfolio [32, 33], but improvements in the products cannot be completely disassociated from parallel changes in the external environment.

As illustrated above, the NNPS comes with strengths as well as limitations. The main strengths of the system include the non-compensatory algorithm, the age-specific nutrient targets and the fact that the system has been applied for more than 10 years across a global product portfolio.

For food products to become more likely to contribute towards nutritionally adequate diets, it is important that they not only contain less of the nutrients to limit, but also more nutrients to encourage [34]. The simultaneous profiling of nutrients to limit and nutrients to encourage has been questioned, as this strategy allows products to achieve better nutrient profiles simply by increasing the content of nutrients to encourage with no change in nutrients to limit [35, 36]. To bypass such a limitation, the NNPS targets for both nutrients to limit and encourage are non-compensatory, an approach also used by other systems including the SAIN, LIM system [37] and Nutrimap [38], ensuring that the NNPS outcome gives a realistic representation of the overall nutritional value of a product and does not simply reflect an increase in nutrients to encourage, for example, through fortification.

The choice of three age groups instead of one is a strength of the proposed system, as it ensures that products for children are designed to support their specific nutritional needs. To differentiate between the needs of younger children and adolescents, two separate age groups were employed, constituting another important improvement compared to previously published systems [34].

For product (re)formulation to address public health nutrition needs, it should combine both public health priorities and the eating habits of local populations. The NNPS was designed for an international product portfolio. In recognition of the importance of addressing local public health priorities, the end user has the possibility to implement local nutritional recommendation or regulation values through the use of a dedicated software, instead of being restricted to the standard values proposed by the system. To further increase the local applicability of the system, data on local consumption habits were used to classify each food product into the most appropriate NNPS food category, e.g., ham was classified as center of plate in France, whereas it is considered under the cold cuts category in Belgium. The same approach was followed to define serving sizes, based on consumer data and local eating habits. Using official recommendations for the setting of nutrient targets and linking the targets directly to DVs is a strength of this system as this is not the case in other nutrient profiling schemes [10].

On the other hand, the main limitations of the NNPS are linked to the need for a large-scale implementation in a varied product portfolio.

One potential use of nutrient profiling systems is to help the food industry to better align its products with public health priorities and goals. The most useful models in this context are category specific (e.g., choices) [12] and therefore more sensitive to food chemistry issues and the specific characteristics of food products. While such systems may appear more permissive, they can help set challenging yet feasible nutrition targets for the reformulation of product categories. The nutrient standards used in the NNPS were saturated fat, added sugar and sodium. Those three nutrients were also used in the creation of the limiting nutrients (LIM subscore) in both the French SAIN, LIM system [37] and the US-based Nutrient Rich Foods Index [39]. Comparative studies have also shown that the LIM subscore was highly correlated with the FSA-Ofcom nutrient profiling model [40], used in the UK to regulate advertising and marketing to children [10]. In the present analyses, we observed reductions in the LIM components as well as small reduction in the total LIM subscore (data not shown). The present nutrient profiling model focuses on saturated fat, sugar and sodium—the same nutrients of concern as identified by regulatory agencies worldwide.

In the context of the capacity of the system to guide effective reformulation, it is apparent that despite it being linked to high levels of reduction in total sugars, the (re)formulation process is not yet complete and further work is needed among specific categories. Similarly, the reformulation of total fat and SFA has been much more category specific and has so far yielded less extensive results. It should also be noted that for the specific case of milk-based beverages, the observed total fat and SFA reduction was mainly achieved by switching from semi-skimmed to skimmed milk as the main ingredient. These differences between nutrients illustrate the simple fact that reformulations occur in waves and efforts are commonly focused on one or two nutrients per wave. Reformulation for the reduction in total fat and SFA, while keeping organoleptic and technical properties intact, is very challenging and new technologies are still being developed to address these issues. This is most likely the reason why among products not meeting all the nutrient targets (classification “NO”) total fat and SFA content were identified as areas for future improvements. At the same time, successful reformulation should also be linked to a taste preference by the consumer, which is not in support of radical changes in nutrient profiles, but it rather encourages gradual improvements in the food supply.

The overall aim of reformulation is to lower excessive consumption of nutrients to limit and—in specific regions—to increase the population intake of nutrients with documented deficiencies. There is a need to assess the potential population impact of the NNPS criteria. Diet modeling, using either existing dietary surveys, Monte Carlo simulation, or diet optimization, could help in determining whether achieving the nutritional targets for each food category does indeed improve the nutritional intake of targeted populations [41–43]. Epidemiological and health economics models may also be useful for assessing the effect of reformulation on a population’s health indicators [1, 3]. For both dietary and economic modeling studies, adequate data with respect to the consumption of manufactured goods would be needed in order to obtain credible estimates of the potential impact of reformulation. In addition, further research across different countries and food categories is necessary to identify the way individuals purchase and prepare their foods.

## Conclusions

The Nestlé Nutritional Profiling System (NNPS) sets meaningful and realistic nutrient targets for nutrition-oriented manufactured food (re)formulation while maintaining consumer preference. It is currently applied across a wide range of food categories in all countries in which Nestlé operates, with the possibility for the end user to adapt targets depending on local conditions, regulations and public health needs. As presented in this study, the application of the NNPS in the USA and France was associated with significant reductions in sodium, total sugars and total fat in the most widely purchased products across eight food categories. Confirmatory analyses are needed to assess the nutritional composition changes resulting from the application of the NNPS in more food categories and in other regions. An estimation of the potential impact on population-wide nutritional intake is needed to validate the methodology and guarantee that the proposed system could help consumers in achieving healthier diets.

## Abbreviations

AS: Added sugars
Ca: Calcium
DV: Daily value
EU: European Union
EFSA: European Food Safety Authority
F: Fiber
FAO: Food and agriculture organization
Fr: Fructose
I&R: Innovation & renovation
IoM: Institute of medicine
Na: Sodium
NCDs: Non-communicable diseases
NNPS: Nestlé Nutritional Profiling System
P: Protein
PUFA: Polyunsaturated fatty acids
RACC: Reference amounts customarily consumed
SFA: Saturated fatty acids
TF: Total fat
TFA: Trans-fatty acids
USDA SR27: United States Department of Agriculture National Database Standard Reference 27
US: United States
USFDA: United States Food and Drug Administration
WHO: World Health Organization
